# Botulinum Toxin Brings a Light to the Shadow of Functional Urology

**DOI:** 10.3390/toxins15050321

**Published:** 2023-05-06

**Authors:** Hann-Chorng Kuo

**Affiliations:** Department of Urology, Hualien Tzu Chi Hospital, Buddhist Tzu Chi Medical Foundation, Tzu Chi University, Hualien 970, Taiwan; hck@tzuchi.com.tw

Functional urology involves a large scale of lower urinary tract dysfunctions (LUTDs), including bladder dysfunctions and bladder outlet dysfunctions. The LUTDs can be neurogenic, inflammatory, or anatomical etiologies in male or female patients, and in elderly or pediatric patients. Currently, we can treat bladder overactivity by antimuscarinics and beta-3 adrenoceptor agonists and manage bladder outlet dysfunction by alpha-blocker, phosphodiesterase inhibitor, and 5-alpha-reductase inhibitors. However, there are still several LUTDs in the shadows of functional urology that are difficult to treat by currently available medications. Botulinum toxin A (BoNT-A) has been approved for treatment of neurogenic detrusor overactivity (NDO) and idiopathic overactive bladder (OAB) refractory to conventional medical therapy [1,2]. In addition to these indications, BoNT-A has been widely used in the treatment of several LUTDs which are not adequately treated by surgical or medical therapies. Currently, the applications of BoNT-A on LUTDs other than NDO and OAB include interstitial cystitis (IC) [3], neurogenic detrusor sphincter dyssynergia (DSD) in patients with spinal cord injury [4], autonomic dysreflexia in high-level spinal cord injury [5], adult non-neurogenic voiding dysfunction such as bladder neck dysfunction, dysfunctional voiding, or poor relaxation of the urethral sphincter [6,7], chronic prostatitis and pelvic pain [8], and pediatric detrusor overactivity and voiding dysfunction [9,10]. However, these indications have not been approved yet, most likely because of the uncertain treatment outcome and adverse events. Due to lack of phase 2 and phase 3 clinical trials, these clinical treatments in functional urology are off-labelled use and cannot be widely applied. Nevertheless, research is still ongoing because the treatment outcome of BoNT-A injections is beneficial for several LUTDs that are difficult to treat by conventional pharmacotherapies or surgical procedures.

In recent decades, clinical and basic research has shown that BoNT-A injections into the detrusor can improve urinary incontinence in elderly patients with OAB but intolerable to adverse events of antimuscarinics. BoNT-A injection into the bladder neck and urethral sphincter can effectively reduce the bladder outlet resistance and facilitate spontaneous voiding in patients with neurogenic or non-neurogenic voiding dysfunction. Repeated intravesical BoNT-A injections have been demonstrated to eliminate bladder inflammation and improve bladder irritative and painful symptoms in patients with IC or ketamine related cystitis. Moreover, clinical trials have shown that BoNT-A encapsulated by liposomes can facilitate BoNT-A protein to penetrate the cell membrane of the urothelium, therefore, the OAB or IC patients might be treated without intravesical injection [11]. Lower energy shock wave can also increase the permeability of urothelial cell membrane and help BoNT-A migrate into the suburothelium of the bladder. These preliminary results provide evidence that the large molecule BoNT-A might be efficiently managed to act on the suburothelial sensory nerves without traumatic injections [12]. In the future, with more clinical studies, we might have a chance to treat LUTDs in functional urology by the device of vehicles to carry BoNT-A into the bladder or urethral tissue without injections. In the shadow of functional urology where conventional medical treatment cannot reach, BoNT-A treatment may bring a light to treat LUTDs.

In this Special Issue, Ou et al. compared the clinical efficacy of BoNT-A injections between young and elderly patients with OAB and analyzed the factors associated with unfavorable outcome. They discovered that the therapeutic efficacy and safety profile of BoNT-A injection are comparable between young and elderly patients with OAB refractory to conventional medication. In the analysis of unfavorable treatment outcome in the elderly patients with OAB, they determined that female gender, presence of diabetes mellitus, and the baseline urodynamic parameters are potential factors. Therefore, before BoNT-A injection for the elderly patients with OAB, the possible treatment outcome should be informed. Hu, et al. reviewed the role of BoNT-A injections for OAB in patients with Parkinson’s disease (PD) and stroke. They determined intravesical injection of 100U of BoNT-A is feasible for patents with PD and OAB to improve incontinence grade with acceptable adverse events of large post-void residual and urinary tract infection, while urethral sphincter BoNT-A injection can be used to treat voiding dysfunction in these patients with OAB due to central nervous lesions. Lee et al. reported the treatment outcome in male patients with OAB after surgery for bladder outlet obstruction. They determined that the therapeutic efficacy of BoNT-A to improve urgency and urinary incontinence is similar with that in female patients with OAB. 

Chen et al. compared the therapeutic effects of urethral sphincter BoNT-A injections in patients with different non-neurogenic voiding dysfunction subtypes. Among the patients with detrusor underactivity, poor relaxation of urethral sphincter, and dysfunctional voiding, patients with dysfunctional voiding benefit most from BoNT-A treatment, both in subjective and objective parameters, and half of patients with detrusor underactivity and poor relaxation of urethral sphincter also had a fair response. A post-void residual volume of >250 mL was a negative predictor in patients with detrusor underactivity. Kao et al. also analyzed the predictive factors for the successful treatment outcome of BoNT-A urethral sphincter injections in patients with different subtypes of voiding dysfunctions. They determined that urethral sphincter BoNT-A injections provide comparative therapeutic efficacy in functional voiding dysfunction and non-neurogenic voiding dysfunction. Among the clinical characteristics of voiding dysfunction, detrusor underactivity and a low voiding efficiency could predict inferior therapeutic outcomes. For patients with NDO, Chen et al. compared the results of clinical treatment outcome between spinal cord injured patients receiving intravesical and urethral sphincter BoNT-A injections. Chow et al. analyzed the therapeutic efficacy of urethral or detrusor BoNT-A injections for patients with autonomic dysreflexia and determined detrusor BoNT-A injections superior to urethral sphincter BoNT-A injections in terms of symptomatic improvement of autonomic dysreflexia.

Five articles in this Special Issue reported the application of BoNT-A in the treatment of IC. Yu et al. reported the treatment outcomes of BoNT-A injections on patients with different subtypes of IC. Pedro Abreu-Mendes et al. reported the long-term real-life follow-up results of intratrigonal BoNT-A injections for patients with IC, and they discovered that intratrigonal BoNT-A injection is effective and durable for IC. Jhang et al. analyzed the therapeutic effects between BoNT-A and platelet-rich plasma (PRP) injections, and determined that BoNT-A injection is superior to PRP injection in reducing bladder pain score. Li et al. reported the results of a new therapy combining BoNT-A injection and Sapylin instillation and discovered significant superior outcome in the mixed group. Finally, Hung et al. reviewed the potential efficacy of intravesical instillation of liposomes and mixed liposomes and BoNT-A in treatment of functional bladder disorders. This treatment might improve LUTDs by BoNT-A without intravesical injection. These five articles provide excellent review and researches on the clinical application of BoNT-A treatment for IC refractory to conventional therapy. Based on previous basic studies of IC, the anti-inflammatory therapeutic effects of BoNT-A might have a chance to combat the inflammation in IC and improve the bladder pain symptoms.

This Special Issue collected original and review articles that focus on the novel applications of BoNT-A in LUTDs in functional urology. The collection of this Special Issue of *Toxins* provides updated knowledge on the current and future position of BoNT-A in the shadows of functional urology and LUTDs.
